# CORRELATES OF MENTAL HEALTH PROBLEMS AMONG OLDER AFRICAN AMERICANS WITH CHRONIC ILLNESS

**DOI:** 10.1093/geroni/igz038.3529

**Published:** 2019-11-08

**Authors:** Eunkyung Yoon, Susie Spence

**Affiliations:** Jackson State University, Jackson, Mississippi, United States

## Abstract

Prior epidemiologic studies have established that high rates of comorbidity of diabetes and depression is common. The aim of this study is to examine prevalence and correlates of mental health conditions among older African Americans with chronic health conditions The sample (n=1,399) from the first round of the NHATS includes older African Americans living in their community: 60% women, 35% married, 39% less than HS, 57% from the South. A two-way MANCOVA was conducted to determine the effect of diabetes and high blood pressures on four DV (no interest, depressed, anxious, worry) while controlling for age. Data indicated that 37% are with diabetes and 80% with high blood pressure and showed that 10% no interest, 4.4% depressed, 3.6% anxious, and 6.4% constantly worry for nearly every day. The main effects of high blood pressure [Wilk’s Λ = .983, F= 2.322, p =.010, η 2 = .015)] and diabetes [Wilk’s Λ = .975, F= 3.526, p =.010, η² = .024] indicate significant effect on combined mental health DV. The covariate significantly influenced the combined MH DV [Wilks’ =.983, F = 3.697, p = .003]. Univariate ANOVA results showed that only anxiety and worry were significantly affected by diabetes and high blood pressure except “depressed” and “no interest” These results partially support that older African Americans with chronic illness are at high risk for general mental health. However, anxiety and worry were more prevalent than just ‘be depressed’. Greater attention should be paid to specific mental expressions of minority older patients.

